# Building a functional multiple intelligences theory to advance educational neuroscience

**DOI:** 10.3389/fpsyg.2013.00950

**Published:** 2013-12-19

**Authors:** Carlo Cerruti

**Affiliations:** Implicit Social Cognition Lab, Department of Psychology, Harvard University, CambridgeMA, USA

**Keywords:** functional multiple intelligences, fMI, multiple intelligences, learning, education, cognitive inhibition, educational neuroscience

## Abstract

A key goal of educational neuroscience is to conduct constrained experimental research that is theory-driven and yet also clearly related to educators’ complex set of questions and concerns. However, the fields of education, cognitive psychology, and neuroscience use different levels of description to characterize human ability. An important advance in research in educational neuroscience would be the identification of a cognitive and neurocognitive framework at a level of description relatively intuitive to educators. I argue that the theory of multiple intelligences (MI; Gardner, 1983), a conception of the mind that motivated a past generation of teachers, may provide such an opportunity. I criticize MI for doing little to clarify for teachers a core misunderstanding, specifically that MI was only an anatomical map of the mind but not a functional theory that detailed how the mind actually processes information. In an attempt to build a “functional MI” theory, I integrate into MI basic principles of cognitive and neural functioning, namely interregional neural facilitation and inhibition. In so doing I hope to forge a path toward constrained experimental research that bears upon teachers’ concerns about teaching and learning.

The nascent field of educational neuroscience challenges scientists to conduct well defined research with relevance to learning processes. However, the fields of education, cognitive psychology, and neuroscience use different levels of description to characterize human ability. In this context it has been relatively difficult to conduct constrained research that remains theory-driven and also maintains its relevance to educators’ complex set of concerns.

To this point, researchers and theorists have set forth broad suggestions about how to build the educational neuroscience community. For example, Fischer et al. (2007) have called for “reciprocal interactions” among neuroscience and education. Researchers have been cautioned to pay more than “lip service” to the different levels of description that characterize the different disciplines comprising educational neuroscience (Anderson and Reid, 2009). Many researchers hope for “bilingual” (Byrnes and Fox, 1998; Mason, 2009) or “multilingual” scholars (Ansari and Coch, 2006) engaged in “bidirectional” work (Ansari, 2005; Ansari and Coch, 2006). Szúcs and Goswami (2007) state that merely “sending information across bridges is not the answer” and instead the field needs “a new colony of interdisciplinary researchers.”

For the neurocognitive research community, an important step beyond these broad suggestions would be the identification of a cognitive framework at a level of description relatively intuitive to educators. If such a cognitive framework exists, then it may be used to shape educators’ questions and concerns into theory-driven, testable neurocognitive research that may advance the education neuroscience field.

In this paper I will suggest that the theory of multiple intelligences (MI; Gardner, 1983), a conception of the mind that motivated a past generation of teachers, may provide cognitive neuroscientists with a framework in which to conduct rigorous educational neuroscience research. However, I will argue that MI prodded teachers to misconstrue the nature of a scientific theory of cognition; teachers took strongly to MI’s value-based claims of the “plurality of intellect,” yet largely failed to recognize that MI did not offer a description of how cognitive processes actually operate nor how an individual child’s mind learns. Finally, I will attempt to integrate into MI basic principles of cognitive and neural functioning, and in so doing I hope to forge a path toward constrained experimental research that bears upon teachers’ concerns about teaching and learning.

## ANATOMICAL MODEL vs. FUNCTIONAL THEORY

Gardner (1993) never intended MI to be applied to education. Though it may come as a surprise to many progressive educators, the MI model was created “not as a program for developing a certain kind of mind or nurturing a certain kind of human being,” Gardner (1993) has written, but rather to explain “the evolution and topography of the human mind.” That is, MI was a map of sorts, seeking to explain what the mind consists of, but not how it works. As such, MI did not address issues critical to the practical and applied needs of educators; beyond recognizing that an intelligence merely exists, MI did not characterize how any one intelligence actually operates, how these intelligences functionally interact with one another, nor how best to teach any one intelligence.

If MI does not, in fact, make any claims about how minds operate nor how to nurture them, what then can explain the affinity educators had for MI immediately upon its introduction in 1983? For space considerations, this question is ultimately beyond the scope of the current analysis. Yet an important clue may come in Gardner’s1987 suggestion that the “real point here,” as he wrote a quarter century ago, “is to make the case for the plurality of intellect.” Gardner was motivated by what he saw as a cultural definition of intelligence that was restricted to verbal and logical–mathematical thought alone. If MI did indeed ride a changing socio-cultural wave in a particular era of history, perhaps this explains teachers’ strong attraction to the pluralistic values MI put forth. MI’s crucial contribution, I believe, was to argue convincingly for the value of kinds of intelligence beyond verbal and logical–mathematical.

Though Gardner considers an intelligence to be an information processing capacity (Gardner and Moran, 2006) MI makes no explicit claims about *how* information is processed. Critically, MI’s value-based claims did not necessarily impact how teachers actually taught nor how they understood the neurocognitive processes of learning. By broadening the definition of intelligence, MI may have been a “catalyzing idea” that “let a hundred flowers bloom” (Gardner, 1995). And yet Gardner (1993, 1999) himself has noted that in many ways MI resembles a Rorschach test, and he credits a colleague with the observation that “MI is popular because it does not come with directions. Educators can say they have adopted it without doing anything differently” (Gardner, 2004). By Gardner’s (1993, 1999, 2006) own reckoning, MI did not at its inception, and never has, made any claim about the actual workings of intelligences.

Why is this a problem of great importance to teachers? In starkest terms, MI simply does not explain how children’s minds learn. Put differently, positing that eight intelligences exist does not characterize in any way how they process information. For example, MI cannot inform a teacher about whether a child’s mathematical–logical intelligence may be nurtured by employing verbal or spatial or kinesthetic intelligence. A teacher with an affinity for MI may indeed view her many students as each having different – and equally valuable – profiles of intelligences. But, critically, this does not provide the teacher insight about whether a child’s mind may benefit from engaging one “relatively independent” (Gardner and Moran, 2006) intelligence to facilitate learning in another intelligence; if the intelligences are indeed largely independent, it may be extremely difficult and inefficient to use kinesthetic intelligence, for example, in an instructional activity that aims to improve verbal intelligence. The main point is that MI simply was not built to explain how the mind works – or how it learns. Yet such knowledge is at the heart of instructional decisions teachers must make.

To scientists, the most pressing problem with Gardner’s model is equally stark: using classical scientific definitions, the theory of MI is not, in truth, a theory. Scientific theories must make falsifiable predictions about thought and behavior (Schacter et al., 2011). Yet MI “makes no claims” (Gardner, personal communication) about how the mind operates or functions, about whether spatial intelligence supports verbal intelligence, for example. With no specific claims, hypotheses or predictions about cognitive processes to make, constrained experimental research is, simply, impossible. With no experimental research that may prove or disprove it, MI may remain only a “catalyzing idea” (Gardner, 1995), though one that I believe had a profound effect on our culture’s views of intelligence and children. Regardless, from a scientific perspective, MI is not a theory. While this claim may appear abstract and of little practical consequence, it is central to my analysis about how to develop MI such that it becomes a proper scientific theory, one that both is generative for the educational neuroscience community and also one that informs teachers’ understanding of learning processes and drives principled, theory-driven instructional decisions.

In sum, two qualitatively distinct propositions have been tremendously conflated in understandings of MI, I argue, a condition that has plagued applications of MI since its inception. On the one hand is a values-based claim, which advocates for making greater efforts to reach the variety of students with different profiles of intelligences that inevitably comprise any teacher’s classroom. And, yet, on the other hand is the need for scientifically and empirically derived claims about how the child’s mind learns. The following distinction is crucial: assigning value to the different intelligences different students exhibit is fundamentally and qualitatively distinct from the scientific enterprise of characterizing how those intelligences work. Teachers may value all kinds of intelligences; knowing how to teach to and develop them is an entirely different, and critical, endeavor.

In this analysis I define MI as a limited “anatomical” map, reflecting Gardner’s (1993) sense that MI was intended to describe the “topography” of the mind. I have belabored the point that MI describes the existence – but not the function – of MI within the mind. In making this distinction I hope to clarify misunderstandings about MI and identify the limits of its scientific reach. Yet in doing so, I hope to advance an argument for how MI may be a suitable framework in which to integrate teachers’ questions and concerns with the experimental research methods of cognitive neuroscience.

## COGNITIVE PSYCHOLOGY: BUILDING A FUNCTIONAL THEORY OF MULTIPLE INTELLIGENCES

The functional MI(fMI) theory I propose focuses on neurocognitive connectivity. A functional theory will build upon an anatomical or structural map by characterizing the patterns of connectivity among relatively autonomous intelligences.

The fMI theory makes two conceptual moves, one qualitative and one quantitative. First, I ask about the quality, or nature, of the interactions between intelligences: *Are the interactions between any two intelligences facilitatory, inhibitory, or neutral?* Second, I ask about the quantity, or strength, of interactions across intelligences: *Is one intelligence relatively more strongly or weakly connected to other intelligences?* These questions are of practical consequence for effective classroom instruction that is focused on how minds learn.

If each intelligence is “relatively independent yet interacting” (Gardner and Moran, 2006) and subserved by specific neurological structures (Gardner, 1998), then a functional theory would predict that any two intelligences can interact in one of three basic ways. In lay terms we would say they may work together, compete, or be indifferent to each other. I will use the terms facilitation and inhibition to describe the former two, indicating that one intelligence can improve the functioning of another, or that one intelligence can impair another. In neurological terms we know that, on the very short timescale at which neurons operate, any two brain regions may be connected such that when one region activates it sends an electrical projection to another region that can excite those downstream neurons, or instead can inhibit, or reduce, the electrical firing of those neurons.

What might be the utility of a functional MI theory centered on a facilitation–inhibition connectivity paradigm? In short, it will help us predict whether an instructional activity largely employing one intelligence is likely to improve, or instead impair, ability in another intelligence. For example, research on the phenomenon known as verbal overshadowing (Schooler and Engstler-Schooler, 1990) has shown that people asked to speak about non-verbal experiences (e.g., face recognition or emotions) often perform more poorly on subsequent tests of memory or analysis [for a review, see Cerruti and Wilkey (2011)]. In one study, young children asked to speak about emotions after watching an emotionally disturbing video performed more poorly on a subsequent learning task compared to a control group (Rice et al., 2007). Given especially how much classroom instruction is verbal, teachers will benefit from understanding when employing verbal cognitive processes helps, and when it hinders, the operations of other cognitive processes. In my experience as a middle school teacher over a decade, I observed that teachers very largely assumed that intelligences facilitate one another, but they did not recognize the real possibility that activity in one part of the mind can in fact inhibit another.

A functional MI theory can also help frame functional and structural neurocognitive experiments. Functional magnetic resonance imaging (fMRI) studies can compare activity in occipital regions of the brain dedicated to visualization when a child verbalizes about a visual geometry problem to a no-verbalization condition. Studies of brain structures may avail themselves of a technology such as diffusion tensor imaging (DTI), which measures fractional anisotropy (FA), thought to be a correlate of the extent of myelination in a region and thus an indicator of speed and efficiency of neural connectivity between two brain regions. Higher FA between two regions known to instantiate the core operations of different intelligences would indicate that those intelligences interact relatively strongly. Then, fMRI studies would need to determine whether these interactions are more facilitatory or more inhibitory.

An fMI theory is very well suited to instructional intervention and longitudinal studies. fMRI and DTI may assess changes in response to an intervention in regional activity, functional connectivity, and FA. For example, these technologies can assess whether intense musical training affects activity in core areas that instantiate numerical cognition, as well as myelination between these areas and core musical brain regions.

Moreover, newer technologies may be of potentially great value for examinations of facilitatory and inhibitory connectivity. Transcranial direct current stimulation (tDCS) sends a very mild electrical current between two electrodes placed on the scalp. Depending on where the electrodes are located, different underlying brain regions will be affected, and in this way specific aspects of cognition can be targeted. In my own work I have found intriguing effects, both facilitatory and inhibitory. For example, anodal stimulation, which increases the propensity for neural firing in the affected region, of left prefrontal cortex improved performance on a verbal task with a high working memory load (Cerruti and Schlaug, 2008). In another study, cathodal stimulation, which blocks or inhibits regional activity, of Broca’s right-hemisphere homolog in fact improved performance on a task of verbal semantic categorization (Cerruti, 2010). Because verbal ability presumably depends relatively strongly on the left hemisphere, this was interpreted as a disinhibition effect: decreased activity in Broca’s right-hemishphere homolog also decreased interhemispheric inhibitory projections, thus permitting increased activity in Broca’s. Studies such as this one reveal the complex functional interconnectivity among the multiple regions of the brain that are invariably involved in complex cognition.

## CONCLUSION

The purpose of a functional theory of MI is to describe how the mind works. The MI framework was not created with the intention of applying it to education (Gardner, 2006), yet educators took strongly to it. In turn, Gardner (1987, 1991) soon took to advocating for MI-inspired environments in schools. In such environments, MI encourages teachers to value and encourage intelligences other than verbal and mathematical. However, MI is incapable of informing teachers about how the individual child’s mind processes information or learns new information.

My analysis has not questioned the anatomical basis of the MI framework. In fact I take as my starting point MI’s assumption that the brain is home to relatively autonomous information processing modules. My approach aims only to detail the interactions of cognitive information processing mechanisms. Such an approach owes much to experimental psychology and neurology, fields that have often been critical of MI (Kornhaber and Gardner, 2006).

My core intention is plain: to advance the utility of MI to both teachers and researchers by building a functional theory of MI. I have argued that as the field of educational neuroscience grows MI may be a particularly useful foundation upon which to build a proper scientific theory of neurocognitive learning processes – one that is at a level of description teachers find to be fairly intuitive. For researchers, a functional theory will help organize experimental research in mind, brain, and education, three disciplines that examine cognition and behavior at different levels of description. For teachers, specification of the functional properties of intelligences will help guide instructional decisions about how a child’s mind learns.

## Conflict of Interest Statement

The author declares that the research was conducted in the absence of any commercial or financial relationships that could be construed as a potential conflict of interest.
